# Lipid Layer Thickness in Soft Contact Lens Wearers With and Without Lenses In Situ—A Systematic Review and Meta-Analysis

**DOI:** 10.3390/jcm15031110

**Published:** 2026-01-30

**Authors:** Maria Sobol, Jacek Pniewski

**Affiliations:** 1 Department of Biophysics, Physiology and Pathophysiology, Medical University of Warsaw, Chałubińskiego 5, 02-004 Warszawa, Poland; 2 Faculty of Physics, University of Warsaw, Pasteura 5, 02-093 Warszawa, Poland; j.pniewski@uw.edu.pl

**Keywords:** tear film, lipid layer, lipid layer thickness, LLT, soft contact lenses, CL

## Abstract

**Objectives:** To systematically review and quantitatively synthesize lipid layer thickness (LLT) measurements in soft contact lens (CL) wearers obtained in the presence and absence of contact lenses in situ. **Methods:** A systematic literature search of PubMed, Scopus, and Web of Science databases was conducted in accordance with PRISMA guidelines. Six studies meeting predefined inclusion criteria were included. Pooled mean LLT values were calculated using fixed-effects models, with heterogeneity, sensitivity analyses, and publication bias assessed. **Results:** In the absence of contact lenses, pooled LLT data from 86 healthy CL wearers yielded a mean LLT of 62.11 nm (95% CI: 47.33–76.90 nm). In the presence of contact lenses, pooled data from 330 subjects demonstrated a mean LLT of 69.52 nm (95% CI: 56.33–82.70 nm). Although LLT values were numerically higher with contact lens wear, the substantial overlap of confidence intervals indicated no consistent or statistically demonstrable difference between conditions. **Conclusions:** This meta-analysis provides the first quantitative synthesis of LLT in CL wearers and highlights the need for standardized methodologies to clarify the clinical relevance of LLT in contact lens-related tear film assessment.

## 1. Introduction

The precorneal tear film constitutes the first refractive surface of the eye and plays a central role in both optical performance and ocular surface homeostasis. By providing a smooth air–tear interface, the tear film minimizes light scatter and higher-order aberrations, thereby contributing to visual quality between blinks [1,2]. Simultaneously, it performs essential biological functions, including lubrication, epithelial protection, and defense against environmental stressors, as summarized in the TFOS DEWS II reports [3,4,5].

The lipid layer, forming the outermost component of the tear film, is a key regulator of these processes. By limiting aqueous tear evaporation and stabilizing the tear film during the interblink period, the lipid layer plays a critical role in maintaining tear film integrity [6,7,8]. Consequently, lipid layer dysfunction is strongly implicated in evaporative dry eye disease and meibomian gland dysfunction [9].

Since 1998, commercial devices, such as Tearscope/Tearscope Plus (Keeler Ltd., UK), EasyTear^®^ VIEW+ (SBM Sistemi, Orbassano, Italy), or Keratograph 5M (Oculus GmbH, Wetzlar, Germany), have been available that allow for the classification of interference patterns of the lipid layer interpreted in terms of so called Guillon patterns or grades, associated with certain thickness ranges [10,11,12].

Advances in non-invasive optical interferometry have enabled in vivo measurements of lipid layer thickness (LLT) in nanometers, leading to its widespread adoption as a quantitative tear film parameter [13]. Commercial systems such as LipiView/LipiView II^®^ and IDRA^®^ estimate the LLT within a nominal range of approximately 15–100 nm and are increasingly used in both clinical practice and research [7,14,15,16]. LLT is therefore often interpreted as a surrogate marker of tear film quality, based on the assumption that a thicker lipid layer confers superior evaporative resistance.

However, growing evidence indicates that LLT is not a simple or linear indicator of “functionally effective tear film”. Experimental studies have shown that increases in LLT do not necessarily lead to proportional reductions in tear film thinning or evaporation, suggesting saturation effects and the importance of lipid organization beyond thickness alone. Clinical studies further demonstrate weak or inconsistent associations between LLT and tear film break-up time, ocular surface staining, or symptoms, even in patients with clinically evident evaporative dry eye [13].

The interpretation of LLT is further complicated by substantial methodological and biological variability. Reported repeatability limits commonly approach ±20–30 nm, indicating that small intergroup differences or longitudinal changes may fall within expected measurement noise [14]. LLT is also influenced by blink behavior, particularly partial blinking, temperature, humidity, age, medication, lifestyle factors, and pathologies, e.g., meibomian gland dysfunction (MGD), as well as by region-of-interest selection and averaging strategy [3,4,5,17,18,19,20]. In general, device-specific characteristics, including proprietary algorithms and upper reporting limits at 100 nm, further limit direct comparability across studies [21].

Recent clinical studies illustrate the context-dependent nature of LLT. Contact lens use is associated with impaired tear film dynamics, including reduced TBUT (tear break-up time), lipid layer thinning, and increased evaporation, often manifesting as ocular discomfort [22,23]. The pre-lens lipid layer (PLLL) over a contact lens is typically thinner and less stable than the natural precorneal lipid layer due to insufficient aqueous support for lipid spreading and poor lens surface wettability, which promotes patch formation [24,25]. Lipid spreading kinetics also differ: whereas adequate aqueous tears allow elastically driven spreading, contact lens-related aqueous deficiency may lead to slower, predominantly viscous behavior [24].

The aim of this study was to provide a comprehensive and quantitative evaluation of LLT in habitual soft contact lens wearers through a systematic review and meta-analysis of the available literature. In light of the limited number of studies, methodological heterogeneity, and inconsistent reporting of LLT outcomes, this study aimed to synthesize the existing evidence and clarify the overall patterns of LLT in CL wearers. The meta-analysis included habitual daily contact lens users and evaluated LLT measurements obtained both in the absence of contact lenses and with contact lenses present in situ.

## 2. Materials and Methods

Studies included in this research were identified through a systematic literature search of the PubMed, Scopus, and Web of Science databases. The literature search and data extraction were conducted manually by the authors, without the use of artificial intelligence, machine learning, or automated screening tools. All stages of study selection, data extraction, and verification were performed by human reviewers. The search was conducted without restrictions on the initial publication date; therefore, all records available in the database from inception to the final search date were considered. Articles published up to and including 9 December 2025 were involved. The inclusion criteria were as follows: original research articles, prospective studies, randomized or cross-sectional studies, human studies, and publications in the English language. Screening was based on the following search terms: (“lipid layer thickness” OR “LLT”) AND (“contact lens” OR “CL”).

**Figure 1:**
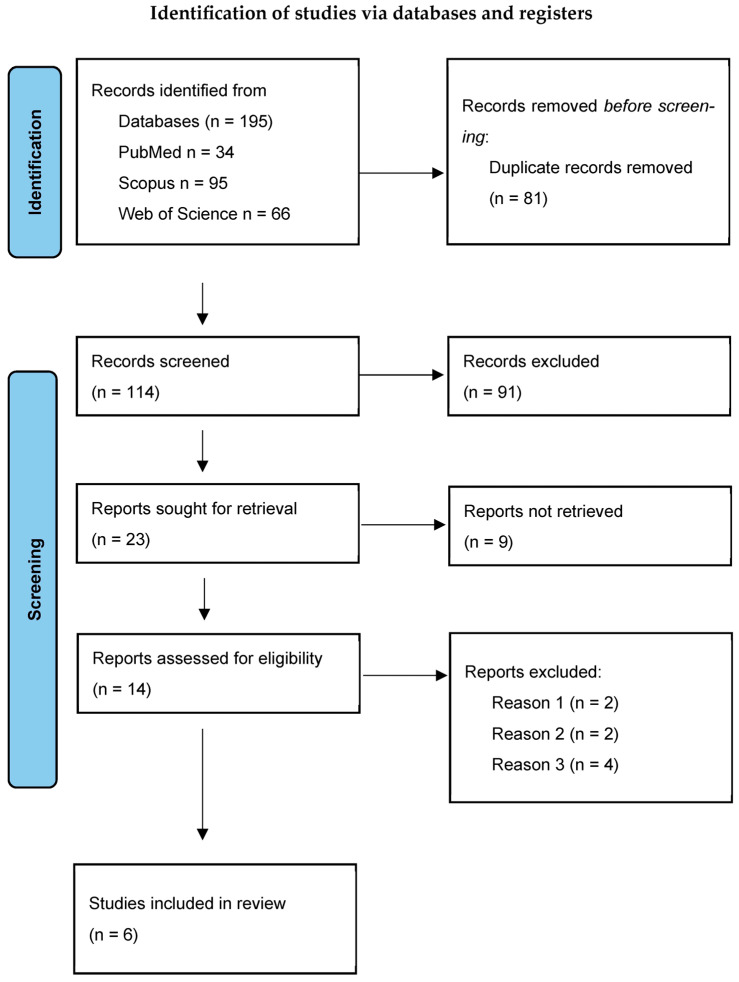
Flow chart for inclusion of studies.

For the analysis, studies were excluded if they (1) reported LLT data only as median and range or median and interquartile range (IQR) or reported LLT without accompanying standard deviation (SD) or confidence interval (CI); (2) used lipid layer thickness Guillon grades to measure LLT or based on an absolute reflectance spectrum. Additionally, (3) studies involving pediatric populations or predominant use of rigid gas permeable (RGP) lenses or orthokeratology lenses were excluded (Figure 1). Case reports, unpublished studies, and conference abstracts were not considered, and the authors of the included studies were not contacted.

Two reviewers (MS and JP) independently screened the titles, abstracts, and full text articles for eligibility based on predefined inclusion and exclusion criteria. Any discrepancies were resolved through discussion and critical reevaluation of the studies until full consensus was achieved. No involvement of a third reviewer was necessary. Only studies for which agreement was reached were included in the final review.

### 2.1. Study Selection

The systematic review was conducted using the PRISMA guidelines [26,27]. A completed PRISMA 2020 checklist is available in the Supplementary Material. Specific requirements are listed below:

#### Study Group

Inclusion criteria: Participants were required to be at least 18 years of age, free from ocular disease or allergies, have a history of contact lens wear, in the case of measurements conducted in the absence of contact lenses, discontinue lens use at least 24 h prior to each study visit, and not be enrolled in any other concurrent clinical studies.

Limits used: Human subject studies published in English.

Timing: No restrictions were applied to the start date of the search. All records available from database inception to 9 December 2025 were eligible for inclusion.

### 2.2. Statistical Analysis

Statistical analysis was performed using the software package TIBCO Software Inc. (Palo Alto, CA, USA) Statistica (data analysis software system), version 13 (www.statsoft.com (accessed on 25 December 2025)). Heterogeneity among studies was assessed using Cochran’s Q test, and the I^2^ statistic was calculated to quantify the degree of heterogeneity (low: 25–50%, moderate: 50–75%, and high: >75%). Since heterogeneity (I^2^ statistics) exceeded 0% for measurements obtained in the absence and presence of contact lenses, a fixed-effects model was applied. Mean LLT values were reported with corresponding 95% confidence intervals (CIs). Forest plots were generated to display individual study estimates and pooled effect sizes with 95% CIs. Sensitivity analyses were conducted by sequentially excluding each study to evaluate the robustness of the findings. Publication bias was assessed using Egger’s and Begg’s tests. In addition, the trim-and-fill method was applied to estimate the number and potential impact of missing studies due to publication bias.

## 3. Results

The search strategy identified 34 articles in PubMed, 95 in Scopus, and 66 in the Web of Science databases. After screening using the phrases lipid layer thickness, LLT, contact lens, and CL, six studies were selected and hence included [28,29,30,31,32,33]. Table 1 includes the demographic and study characteristics of the participants, such as age, sex, number of subjects, eye selection for analysis, contact lens type (when reported), duration of contact lens wear, and the device used.

Most subjects included in both meta-analyses were current contact lens (CL) wearers who used contact lenses at least 5 days per week for a minimum of 6 h per day. In the study by Kim et al., the duration and frequency of contact lens use were not clearly specified; the only information provided was that participants had worn contact lenses in the past [32]. Contact lens discomfort (CLD) symptomatology was assessed using the Contact Lens Dry Eye Questionnaire (CLDEQ-8) in the studies by Valencia-Nieto et al. [29,30], Novo-Diez et al. [31], and Kumar et al. [28]; Tichenor et al. [33] used a longer version of this questionnaire, whereas in the study by Kim et al. [32], participants completed a general questionnaire regarding ocular health. Subjects with active ocular surface disease, including corneal infection, allergy, corneal edema, or iritis, were excluded. Additional exclusion criteria included the use of topical ophthalmic medications, the presence of neurological disorders (e.g., epilepsy), use of systemic medications known to affect tear film dynamics, and pregnancy or lactation. Two of the included studies were conducted in the United States, one in Australia, and three in Spain.

LLT was measured using either the LipiView Ocular Surface Interferometer (TearScience Inc., Milpitas, CA, USA) or the LipiView II Interferometer (Johnson & Johnson Vision, Santa Ana, CA, USA).

**Figure 2:**
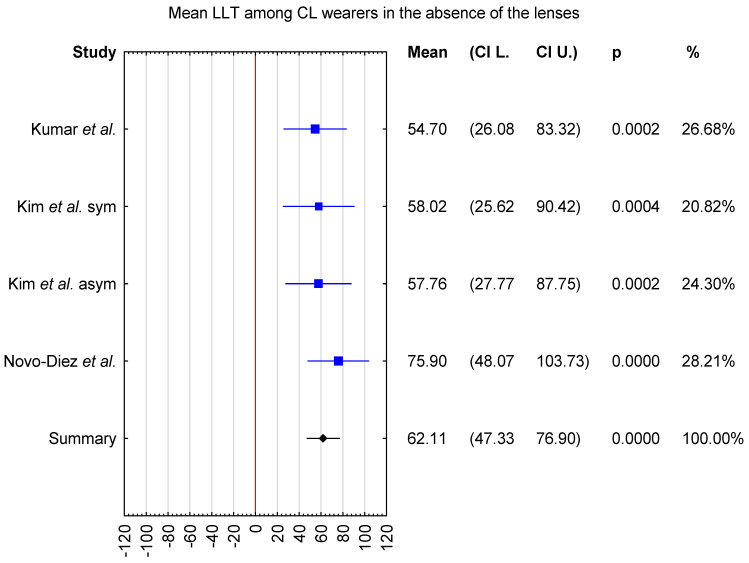
Forest plot of mean LLT among contact lens wearers, measured in the absence of the lenses, with *p*-value indicating level of statistical significance [28,31,32]. The size of the box represents the point estimate for each study in the forest plot and is proportional to that study’s weight-estimate contribution to the summary estimate. Horizontal lines represent 95% CI.

In the first meta-analysis, the mean LLT was evaluated in individuals who wore contact lenses daily, with measurements obtained in the absence of contact lenses. Three publications were included in this analysis. Additionally, in the study by Kim et al. [32], participants were classified into two subgroups, symptomatic and asymptomatic contact lens wearers, based on scores from the CLDEQ-8 questionnaire. A total of 86 subjects were included in the analysis; the majority were female (n = 70, 81.4%), while 16 were male. The mean age was 24.9 years (95% CI from 20.9 to 29.0 years). The sample size of the individual studies ranged from 20 to 24 participants. In the studies by Kumar et al. [28] and Novo-Diez et al. [31], the data from a single eye randomly selected for each participant were analyzed, and Kim et al. [32] analyzed data exclusively from the right eye. As a result, the mean LLT was 62.11 nm with CI levels ranging from 47.33 to 76.90 nm (Figure 2).

**Figure 3:**
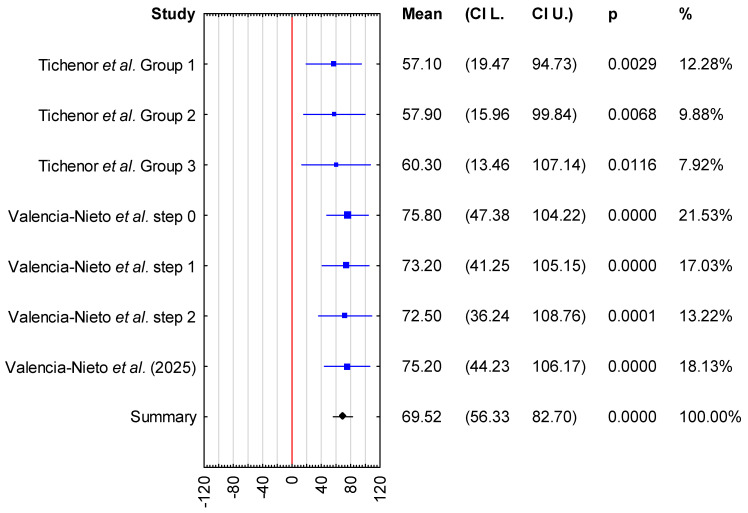
Forest plot of mean LLT among contact lens wearers, measured in the presence of the lenses, with *p*-value indicating level of statistical significance [29,30,33]. The size of the box represents the point estimate for each study in the forest plot and is proportional to that study’s weight-estimate contribution to the summary estimate. Horizontal lines represent 95% CI.

In the second meta-analysis, mean LLT was evaluated in individuals who wore contact lenses daily, with measurements obtained in the presence of contact lenses. Three publications were included in this analysis. In the study by Tichenor et al. [33], participants were allocated to three intervention Groups: Group 1 used the Bruder moist heat compress twice daily, Group 2 used the compress once daily in the evening, and group 3 used a warm washcloth twice daily. In addition, Valencia-Nieto et al. [30] stratified participants according to the Tear Film and Ocular Surface Society (TFOS) classification of contact lens discomfort (CLD) progression, as well as a simplified classification proposed by the authors. For the purposes of the present meta-analysis, the author-defined grouping was adopted, in which TFOS steps 1 and 2 and steps 3 and 4 were combined. Data included in the meta-analysis were obtained from baseline measurements; however, the original group stratifications defined in the included studies were maintained. A total of 330 participants were included in the analysis; the majority were female (n = 229, 69.4%), while 101 were male. The mean age was 32.9 years (95% CI: 23.6–42.3 years). The sample sizes of the individual studies ranged from 51 to 142 participants. In the study by Valencia-Nieto et al. [30], data from a single eye randomly selected for each participant were analyzed, whereas Tichenor et al. [33] analyzed data exclusively from the right eye. The pooled mean LLT was 69.52 nm, with a 95% confidence interval ranging from 56.33 to 82.70 nm (Figure 3).

In Figure 2, LLT values reported by Kim et al. were presented separately for symptomatic and asymptomatic contact lens wearers, allowing the visualization of subgroup-specific trends. In Figure 3, participants were displayed according to their original randomization into Groups 1, 2, or 3, as defined by Tichenor et al., including differences in the frequency of Bruder moist heat compress use. As shown in the forest plot, baseline LLT values were comparable across the classified groups, indicating no meaningful differences between groups at the start of the studies.

Furthermore, the inclusion of study weights and confidence intervals in the forest plots enables the assessment of inter-study heterogeneity and the relative contribution of individual studies to the pooled estimates, thereby improving the accessibility and interpretability of variable behavior and overall trends in LLT outcomes.

### 3.1. Risk-of-Bias Assessment

Overall, the forest plot for the mean LLT in individuals who wore contact lenses daily, with measurements obtained in the absence of contact lenses, showed that the highest mean LLT was reported in the study by Novo-Díaz et al. [31]. The confidence intervals for the studies by Kumar et al. [28] and Kim et al. [32] ranged from 26.08 to 90.42 nm, whereas the confidence interval reported by Novo-Diez et al. [31] ranged from 48.07 to 103.73 nm. In the presence of contact lenses, the lowest mean LLT was observed in the study by Tichenor et al. [33]. The confidence intervals for LLT ranged from 13.64 to 107.14 nm in Tichenor et al. [33] and from 36.24 to 108.76 nm in Valencia-Nieto et al. [30].

**Figure 4:**
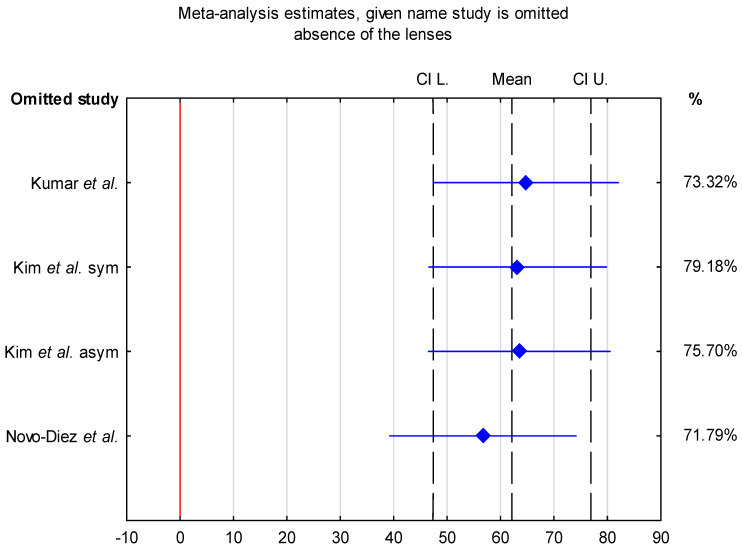
Sensitivity analysis of individual studies on the pooled mean LLT with 95% confidence intervals [28,31,32]. Each study listed on the Y-axis was sequentially omitted to evaluate its impact on the overall pooled estimate (contact lens absence).

**Figure 5:**
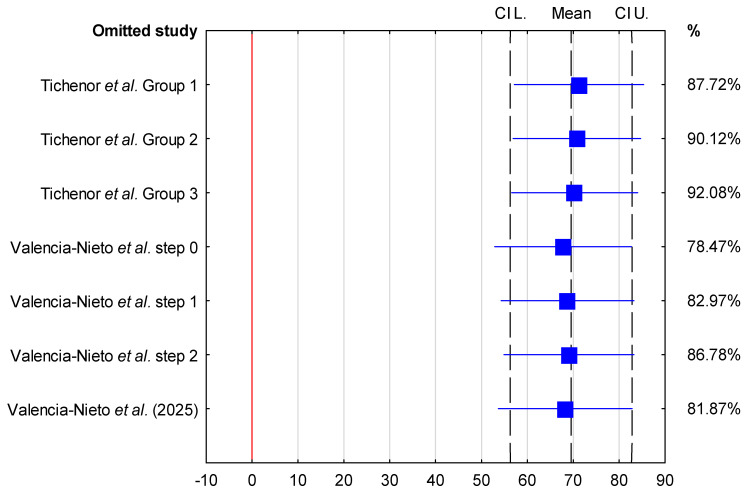
Sensitivity analysis of individual studies on the pooled mean lipid layer thickness (LLT) with 95% confidence intervals [29,30,33]. Each study listed on the Y-axis was sequentially omitted to evaluate its impact on the overall pooled estimate (contact lens presence).

Sensitivity analysis demonstrated that the pooled mean LLT in the absence of contact lenses varied from 56.7 nm (95% CI: 39.25–74.14 nm) when the study by Novo-Diez et al. was excluded to 64.81 nm (95% CI: 47.55–82.08 nm) when the study by Kumar et al. was excluded. In the presence of contact lenses, the pooled mean LLT ranged from 67.8 nm (95% CI: 52.91–82.68 nm) following the exclusion of step 0 data from Valencia-Nieto et al. to 71.26 nm (95% CI: 57.18–85.34 nm) when the study by Tichenor et al. was excluded. These findings indicate that the pooled mean LLT estimates were stable and not disproportionately influenced by any single study (Figure 4 and Figure 5).

### 3.2. Trim-and-Fill Method

**Figure 6:**
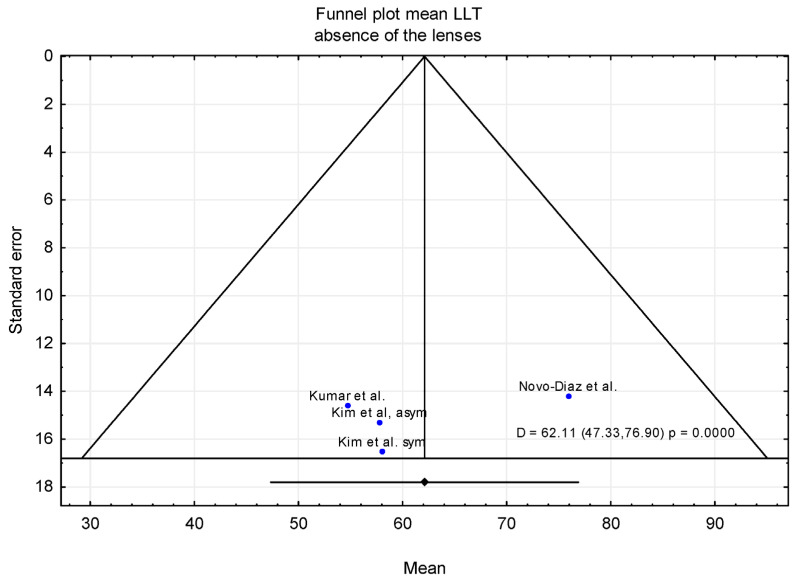
Funnel plot before (contact lens absence) [28,31,32].

**Figure 7:**
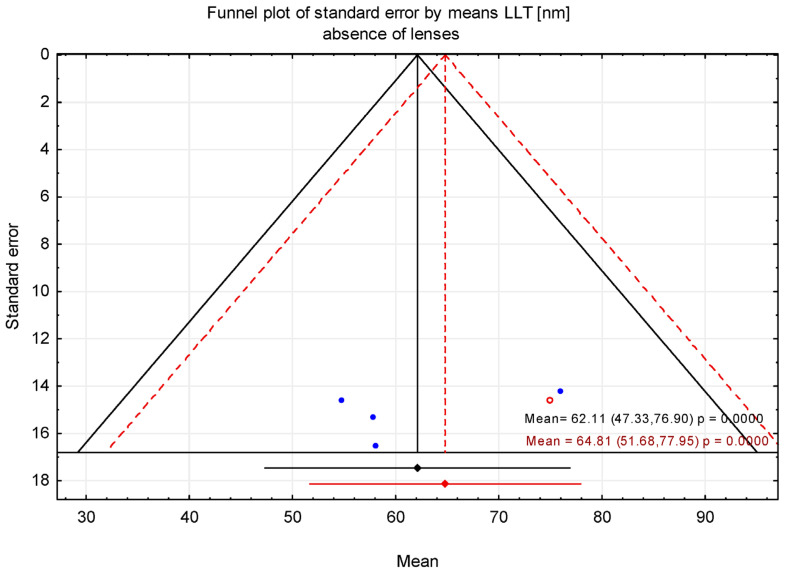
Funnel plot before and after applying the trim-and-fill method (contact lens absence). Blue circles represent observed (real) studies, while red circles represent imputed (“filled”) hypothetical studies.

**Figure 8:**
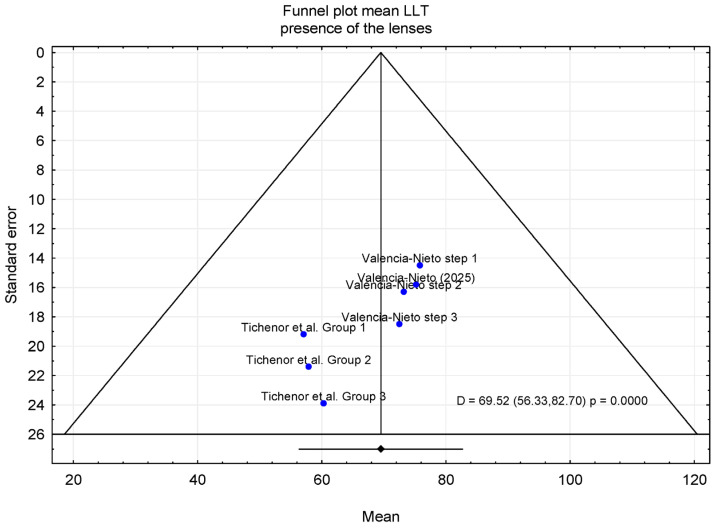
Funnel plot before (contact lens presence) [29,30,33].

**Figure 9:**
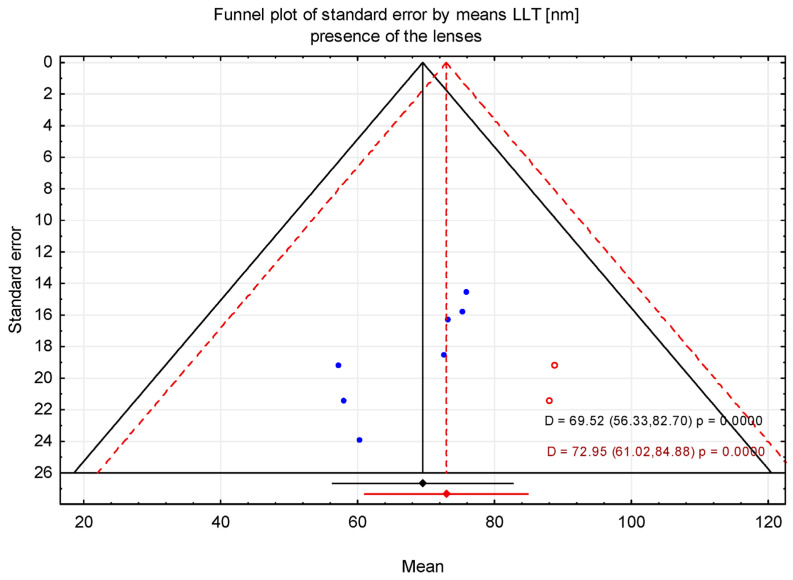
Funnel plot before and after applying the trim-and-fill method (contact lens presence). Blue circles represent observed (real) studies, while red circles represent imputed (“filled”) hypothetical studies.

The trim-and-fill method was applied to assess the potential impact of publication bias suggested by asymmetry in the funnel plot. After adjustment using the trim-and-fill procedure, the estimated mean LLT was 64.81 nm (95% CI: 51.68–77.95 nm) in the absence of contact lenses and 72.95 nm (95% CI: 61.02–84.88 nm) in the presence of contact lenses (Figure 6, Figure 7, Figure 8 and Figure 9). Figure 6 shows the funnel plot of mean LLT in the absence of the contact lenses. Figure 7 shows the funnel plot of the standard error by mean LLT in the absence of the contact lenses. Figure 8 shows the funnel plot of mean LLT in the presence of the contact lenses. Figure 9 shows the funnel plot of the standard error by mean LLT in the presence of the contact lenses.

Furthermore, the results of Egger’s test (*p* = 0.472 for the absence of the lenses and *p* = 0.017 for the presence of the lenses) indicated minimal potential for publication bias in studies without contact lenses, whereas studies with contact lenses suggested possible publication bias. These findings were consistent with Begg’s test (*p* = 0.998 and *p* = 0.024, respectively).

## 4. Discussion

Lipid layer thickness represents an important component of tear film dynamics in contact lens wearers, contributing to tear film stability and evaporative control. Reduced LLT has been associated with increased tear evaporation, tear film instability, and evaporative dry eye, all of which may contribute to discomfort and reduced contact lens tolerance [1,6]. In CL users, the disruption of the lipid layer may be further influenced by the mechanical interaction between the lens and the eyelids, lens material and surface properties, and meibomian gland function [22]. Consequently, the assessment of LLT can provide valuable insight into tear film alterations associated with contact lens wear, but it should be interpreted in conjunction with other clinical parameters rather than as an isolated marker of tear film quality.

To the best of our knowledge, this study represents the first meta-analysis to quantitatively synthesize interferometrically measured LLT values in contact lens wearers, with measurements obtained both in the presence and absence of contact lenses. Previous studies addressing LLT in CL users have generally involved limited sample sizes and heterogeneous methodologies, restricting the ability to establish robust reference values. By pooling the available data from eligible studies, the present analysis provides an initial quantitative framework for interpreting LLT measurements in this population including contact lens use (presence vs. absence) and demographic factors (age and sex).

To obtain more robust estimates, we conducted a systematic literature search of PubMed, Scopus, and Web of Science. This approach enabled the inclusion of a relatively large cohort of healthy individuals aged 18 years or older with a history of CL wear. Participants were free from ocular disease or allergies, and individuals using topical ophthalmic medications, those with neurological disorders (e.g., epilepsy), and those taking systemic medications known to affect tear film dynamics, as well as pregnant or lactating individuals, were excluded.

Several studies were excluded during the selection process to ensure methodological comparability. In particular, investigations relying on color-based grading systems rather than continuous nanometer-scale LLT measurements were excluded, as these approaches are not directly comparable with interferometric devices reporting quantitative LLT values. Similarly, studies with very small or heterogeneous control groups were omitted to reduce bias and improve the interpretability of pooled estimates. These exclusions were based on methodological considerations rather than study quality per se and reflect the current lack of standardization in LLT assessment.

Publication bias analyses yielded differing results depending on the measurement conditions. For LLT measured in the absence of contact lenses, both Egger’s (*p* = 0.472) and Begg’s (*p* = 0.998) tests suggested a low risk of publication bias. In contrast, analyses involving measurements obtained with contact lenses in situ indicated possible small-study effects (Egger’s *p* = 0.03, Begg’s *p* = 0.025). However, these findings should be interpreted cautiously, as the limited number of available studies reduces the reliability of formal bias detection methods and increases susceptibility to random effects.

To further assess the potential influence of publication bias, trim-and-fill analyses were performed. The adjusted pooled mean LLT estimates were comparable to the unadjusted values. However, the robustness of this method is constrained by the small number of studies included in each analysis (n = 3). Funnel plot-based approaches and imputation techniques such as trim-and-fill are known to be less reliable when few studies are available. Nevertheless, the consistency of results across sensitivity analyses suggests that publication bias is unlikely to have materially affected the overall conclusions of this meta-analysis.

The eligible study by Kathryn S. Park et al. [34] was excluded because the control group consisted of only 10 participants, three of whom were spectacle wearers, introducing heterogeneity in visual correction status and limiting the suitability of the control data for meta-analysis. Additionally, the study by Dutta et al. [35] was also excluded because it reported bilateral eye data and enrolled non-contact lens wearers, including participants with a history of contact lens wear within the preceding six months, which did not meet the predefined inclusion criteria for contact lens–LLT analysis.

In contrast, data from Mukesh Kumar et al. [28] were included in the meta-analysis using the reported mean LLT values, as the primary aim of that study was to assess the repeatability of LLT measurements obtained with the LipiView interferometer following daily disposable contact lens wear. The reported mean LLT values were therefore considered appropriate for inclusion in the pooled analysis. The study by Young Hyun Kim et al. [32] presented LLT measurements obtained at multiple visits in the same cohort of participants, both in the presence and absence of soft contact lenses (SCLs). Specifically, LLT measurements were performed during visits without SCL wear (visits 2 and 3) and during visits with SCL wear (visits 3 and 4). However, ambiguity in the reporting of LLT values for later visits (visits 4 and 5), combined with uncertainty regarding CL wear status and the inclusion of repeated measurements from the same population, led to the exclusion of these data from the pooled analysis to avoid duplication and misclassification.

### 4.1. Perspectives on Contact Lens Development, Surface Modifications, and Complementary Therapies

The present meta-analysis indicates that LLT does not consistently differ between measurements obtained in the presence and absence of contact lenses. This finding highlights a key limitation of LLT as a standalone biomarker and underscores the need to interpret LLT within the broader context of contact lens material properties, surface modifications, and adjunctive therapeutic strategies.

Recent developments in contact lens design increasingly focus on optimizing lens–tear film interactions rather than altering bulk material properties alone [36,37,38]. Modern silicone hydrogel lenses incorporate advanced surface treatments and water-gradient technologies aimed at improving wettability and tear film stability. However, recent evidence suggests that such innovations may primarily influence lipid layer organization and spreading behavior rather than inducing measurable increases in mean LLT. Additionally, it emphasizes that tear film functionality depends on lipid layer uniformity, interblink stability, and dynamic redistribution, factors not fully captured by static LLT measurements alone [4,13,17,18,20].

Plasma treatments, hydrophilic coatings, and biomimetic surface designs have been shown to reduce tear film disruption and promote more homogeneous pre-lens lipid layer formation [37].

Complementary therapies further illustrate the contextual nature of LLT. Recent studies demonstrate that interventions targeting meibomian gland function, including thermal eyelid therapies and lipid-containing ocular lubricants, can alleviate contact lens discomfort and evaporative dry eye symptoms [39,40].

Taken together, these observations highlight the need for future investigations to move beyond mean LLT values toward multidimensional assessments of tear film behavior. Integrating LLT with dynamic, spatial, and functional tear film metrics may better reflect the clinical impact of emerging contact lens technologies and adjunctive treatments.

### 4.2. Limitations of the Study

Several limitations of this meta-analysis should be acknowledged. First, the number of studies and participants contributing to the pooled LLT estimate without contact lenses was relatively small, which may limit precision and generalizability. Second, substantial heterogeneity existed across studies with respect to contact lens materials, surface properties, wearing schedules, and duration of lens use, precluding meaningful subgroup analyses. Additional sources of variability include differences in measurement protocols, environmental conditions, blink behavior, and device-specific algorithms, all of which may influence reported LLT values.

Furthermore, subgroup meta-analyses based on symptom status, sex, or age could not be performed due to the insufficient reporting of these variables. Only one included study stratified participants according to symptomatic and asymptomatic contact lens wear status, and none reported LLT outcomes separately by sex. In addition, the relatively narrow and comparable age ranges across studies limited the ability to evaluate age-related differences. Table 2 shows the possible influence of the variables, such as lens and wear characteristics, age, and protocol, on LLT.

Standardized and comprehensive reporting of demographic and clinical characteristics in future studies is therefore essential to enable robust subgroup analyses and to clarify the potential influence of these factors on LLT.

Despite the limitations, the present analysis of LLT provides valuable insight into tear film dynamics in contact lens wearers. Our meta-analysis revealed a pooled mean LLT of 62.11 nm (95% CI: 47.33–76.90 nm) in the absence of contact lenses and 69.52 nm (95% CI: 56.33–82.70 nm) in the presence of lenses, with the overlapping confidence intervals indicating no consistent or statistically demonstrable change due to lens wear. Nonetheless, the LLT remains clinically informative, as variations in lens material, surface properties, and wearing schedules can influence lipid deposition and tear film stability. In symptomatic wearers, a reduced LLT may highlight tear film compromise and guide targeted interventions, including the modification of the lens type, implementation of meibomian gland-directed therapies, or use of lipid-containing ocular lubricants. Therefore, LLT assessment, when interpreted alongside other clinical metrics such as tear break-up time and ocular surface staining, can support strategies to optimize contact lens comfort, visual stability, and long-term tolerance.

## 5. Conclusions

This meta-analysis provides the first quantitative synthesis of lipid layer thickness (LLT) in habitual soft contact lens wearers, incorporating measurements obtained both in the presence and absence of lenses in situ. Although LLT values were numerically higher when measured with contact lenses in situ, the substantial overlap of confidence intervals indicates that lens wear does not consistently or significantly alter the LLT. Nonetheless, several limitations, including small sample sizes, methodological heterogeneity, and limited subgroup reporting, underscore the need for larger, standardized studies. Future research should integrate LLT with dynamic and functional tear film metrics, account for demographic and clinical subgroups, and employ consistent interferometric measurement protocols to better guide contact lens design, clinical management, and therapeutic interventions. In conclusion, while the LLT provides valuable insight into tear film alterations in contact lens wearers, it should be interpreted alongside other clinical assessments to optimize comfort, visual stability, and long-term lens tolerance.

## Figures and Tables

**Table 1:** Base characteristics of the included studies.

Ref. #	Age Mean ± SD Range	Number of Subjects	Eye	CL Type	Duration of CL Wear	LLT Mean ± SD [nm]	Study Design	Device
Kumar et al., 2025 [28]	25.4 ± 6.1 16 F 4 M	20	random	Clariti 1 day (CooperVision) somofilcon A Precision 1 (Alcon) verofilcon	experienced CL users +30 days in the study (min. 5 d/week 6 h/day)	baseline: 54.7 ± 14.7 95% CI: 50.1–59.2 somofilcon: 53.3 ± 15.9 95% CI: 45.1–61.5 verofilcon: 52.7 ± 14.5 95% CI: 45.3–60.3	Prospective	LipiView II
Valencia-Nieto et al., 2025 [29]	33.1 ± 11.9 87 F 50 M	137	random	soft CLs	min. 1 year	75.2 ± 15.8 min-max: 36.0–100.0	cross-sectional, observational study	LipiView II
Kim et al., 2023 [32]	SYM 23.5 ± 3.8 ASYM 22.7 ± 3.8 range 18–34 36 F 6 M *	42 (21 SYM, 21 ASYM)	right	soft CLs comfilcon A (CooperVision)	SYM: 8.2 ± 3.9 yrs ASYM: 7.7 ± 3.9 yrs	SYM (visits 2–3, no-lens) 58.02 ± 16.53 ASYM (visits 2–3, no-lens) 57.76 ± 15.3 SYM (visits 4–5, no-lens) 74.95 ± 15.10 ASYM (visits 4–5, no-lens) 70.12 ± 15.17		LipiView
Valencia-Nieto et al., 2024 [30]	34.4 ± 12.6 97/92 F † 53/50 M	150/142	random	soft CLs	days/weeks: 5.2 ± 2.1 hours/day: 9.0 ± 3.7	Step 0: 75.8 ± 14.5 Step 1: 73.2 ± 16.3 Step 2: 72.5 ± 18.5	prospective, cross-sectional study	LipiView II
Tichenor et al., 2019 [33]	Group 1: 32 ± 8 16 F 1 M Group 2: 32 ± 12 17 F Group 3: 36 ± 11 17 F	51	right	soft CLs	Group 1: 16.0 ± 6.9 yrs Group 2: 14.5 ± 7.9 yrs Group 3: 13.6 ± 8.8 yrs	Group 1: 57.1 ± 19.2 Group 2: 57.9 ± 21.4 Group 3: 60.3 ± 23.9	single-center, randomized, open-label, unmasked clinical trial	LipiView II
Novo-Diez et al., 2025 [31]	23.3 ± 3.9 18 F 6 M	24	random	omafilcon A (hydrogel) stenofilcon A Si-Hy (CooperVision)	8.5 ± 5.3 yrs 5.4 ± 1.9 days/week 8.5 ± 3.1 h/day	baseline: 75.9 ± 14.2 hydrogel: 64.9 ± 15.5 Si-Hy: 75.8 ± 14.0	randomized crossover, double-masked study	LipiView II

* SYM/ASYM—symptomatic/asymptomatic wearers. † Initially, 150 participants were recruited, but 8 RGP lens wearers were removed from the final analysis.

**Table 2:** Possible influence of the variables on LLT.

Variable	Measurement/Category	Observed Effect on LLT	Interpretation
Lens wear	Presence vs. absence of contact lenses	Mean LLT slightly higher with lenses but CI overlap indicates no consistent effect	Lens presence alone does not reliably alter LLT, mechanical/lipid interactions may be subtle
Lens material and surface	Soft CLs, silicone hydrogel, surface coatings, water-gradient technology	Advanced surface treatments may improve lipid spreading, not necessarily mean LLT	Influences tear film uniformity, interblink stability, and comfort
Duration of wear	Hours/day (≥6 h), days/week (≥5)	No consistent correlation detected, small sample sizes limit conclusions	Longer wear may affect lipid deposition, but data insufficient
Symptom status	Symptomatic vs. asymptomatic CL wearers	Symptomatic wearers tended to have lower LLT	Supports relationship between reduced LLT and CL discomfort/tear instability
Age	Mean 24–34 years	No trend detected	Narrow age range, unable to assess age-related differences
Sex	Female vs. male	Not consistently reported	Possible influence via meibomian gland function, insufficient data
Measurement protocol	Eye selection (right eye/random), device (LipiView, LipiView II), baseline vs. follow-up	Small variations across studies, pooled sensitivity analysis showed stable mean LLT	Differences in protocols introduce heterogeneity but do not materially affect pooled estimates

## Data Availability

All articles included in the meta-analysis are accessible through the databases of their respective publishers. All results derived from the meta-analysis are explicitly reported in the article.

## Supplementary Materials

The following supporting information can be downloaded at: https://www.mdpi.com/article/10.3390/jcm15031110/s1, PRISMA 2020 Checklist.
